# A single component landing site for efficient transgenesis using recombination-mediated insertion

**DOI:** 10.17912/micropub.biology.001784

**Published:** 2025-09-02

**Authors:** Aiden A. Beck, Michael L. Nonet

**Affiliations:** 1 Dept. of Neuroscience, Washington University Medical School

## Abstract

Recombination-mediated insertion (RMI) is a recently developed method for creating *C. elegans* transgenes using phiC31-mediated recombination. RMI was developed as a two-component approach that relies on an *att* landing site and an unlinked source of phiC31 and FLP recombinases. Here, we describe both a landing site that incorporates the recombinase cassette and matching targeting vectors. The new landing site is located at a well-vetted MosSCI insertion on Chr IV and injections of single animals typically yield multiple independent insertions. We document the utility of this RMI landing site by creating 14 fluorescent reporters for pharyngeal gland cells.

**Figure 1. Overview of the methodology and efficiency of single-copy transgene integration using the new RMI landing site.**
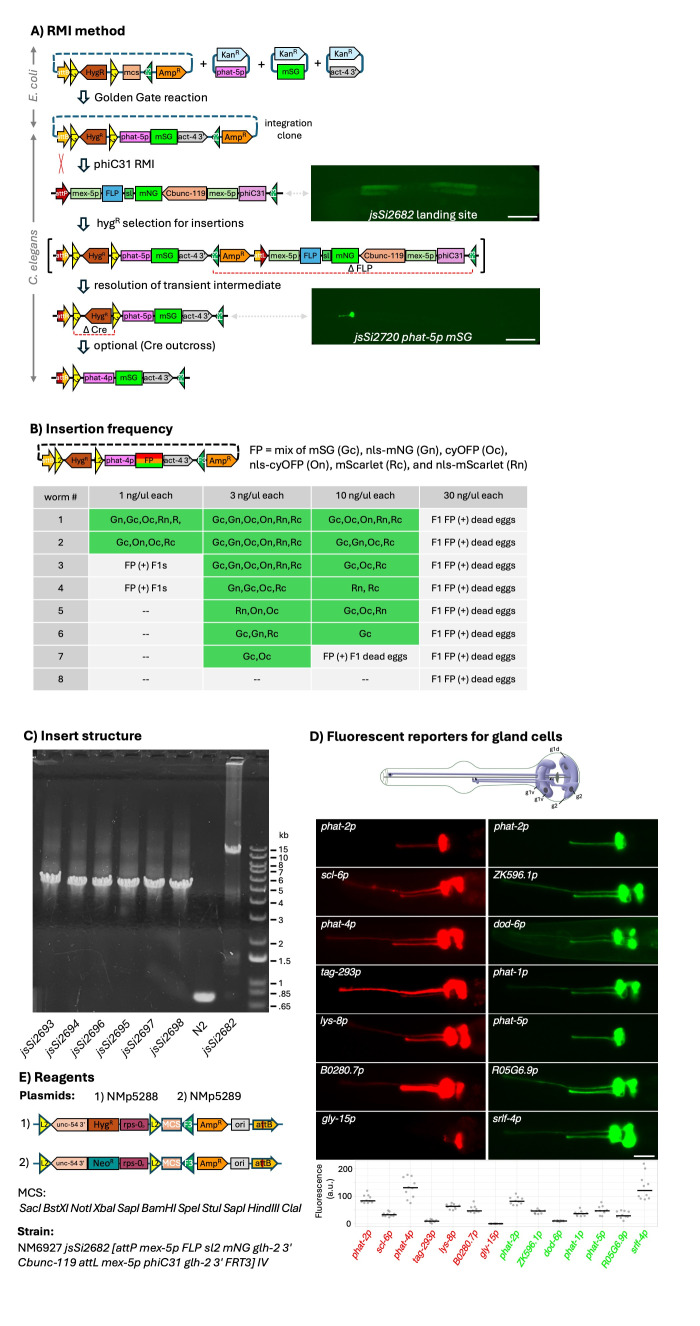
A) Schematic of the RMI procedure. An integration clone is constructed using standard molecular cloning techniques. We recommend using a Golden Gate cloning approach as shown here. The integration clone is injected into the RMI landing site strain and insertions are selected for by the addition of hygromycin (or neomycin) 3 days after injection. The insertions occur by phiC31-mediated recombination between the chromosomal *attP* site and the plasmid *attB* site. This creates an transientsimply intermediate which is resolved by FLP recombinase excising the germline recombinase cassette. Homozygous inserts can be distinguished from heterozygotes by the absence of the germline mNG signal of the landing site as shown in the L4 images of the landing site strain and the homozygous insertion strain. This germline signal is dim until late L4 and become progressively strong as the worms age. Note that the orientation of the landing site on chromosome IV is such that transcription of recombinases is towards bp 1 of the chromosome. It is shown here in the opposite orientation for clarity. B) Table showing the RMI insertion frequency from individually injected worms. Each worm was injected with a mix of 6 integration clones at the listed concentration all containing the *phat-4* gland cell promoter driving a fluorescent protein. The frequency of different FP insertions varied with one to three different types being common and the other being less abundant. (--) indicates no fluorescent progeny were observed. C) Assessment of the structure of insertions. Six distinct transgenes (isolated from progeny of worm #1, 3 ng/ul injection Fig. 1B) each expressing a different FP were analyzed. See Extended Data Table for sequence of insertions. DNA agarose gel of PCR products obtained from amplifying genomic DNA from the homozygous insertions using oligonucleotides on either side of the insertion. The DNA ladder is shown on the right. D) Demonstration of the robustness of RMI. Images of the pharynx of fourteen different insertions created using a set of 13 different gland cell promoters. On the top of the image is a schematic of the five pharyngeal gland cells adapted with permission from the handbook of *C. elegans* Anatomy (Altun and Hall, 2020). The intensity of the images was adjusted to enable visualization of the gland cell process of the low expressing cells in transgenes which expressed at distinct levels in different subsets of gland cells. Green transcriptional fusions are to mStayGold and red fusions are to mScarlet-I3. The strength of expression from each transgene was quantified by plotting the integrated intensity of each transgene in the posterior pharyngeal bulb where all the gland cell somas reside. The red and green signals can be roughly compared because the signal intensity of the red and green signals were normalized to the common *phat-2 p* FP transgenes. E) Reagents described herein available for performing RMI. The *C. elegans* strain is available from the *Caenorhabditis* Genetics Center and the plasmids are available from Addgene. Scale Bars: A; 100 µm D; 20 µm. Abbreviations: FP, fluorescent protein; MCS, multiple cloning site; sl2, *gpd2-3* sl2 *trans* splicing region; L2, *lox2722* ; F3, *FRT3* ; nls, nuclear localization sequence; mSG, mStayGold; nls-mNG, nls-mNeonGreen; cyOFP, cyan-excitable Orange FP.

## Description

Transgenic animals are powerful tools for the study of basic biological processes in many model organisms. In recent years, we developed several techniques to facilitate creating transgenes in *C. elegans* . First, we developed recombination-mediated cassette exchange (RMCE), an efficient FLP recombinase-based method for creating single copy insertions (Nonet, 2020, Nonet, 2023). In addition, we expanded and optimized bipartite reporter systems for *C. elegans* (Nonet, 2020, Knoebel et al., 2025). To simplify genetics using bipartite reporter systems, it was desirable to link the driver and reporter into a single chromosomal locus. Thus, we also developed recombination-mediated homolog exchange (RMHE) to recombine *attP* -tagged reporters and *attB* -tagged driver elements using phiC31 recombinase (Nonet, 2024). Alongside, we developed a recombination-mediated insertion (RMI) approach to efficiently integrate whole plasmids into genomic *attB* or *attP* tagged driver and reporter transgenes (Nonet, 2024). However, RMI in worms was developed in conjunction with RMHE. Since RMHE is performed using crosses rather than injections, both RMI and RMHE were developed as two-component methods using an unlinked source of recombinases. Here we now describe a novel single component RMI landing site that incorporates the recombinases into the landing site. This enables the creation of novel single-copy transgenic lines in 6-8 days.

RMI requires both phiC31 integrase and FLP recombinase to efficiently integrate DNA (Nonet, 2024). The phiC31 integrase catalyzes the genomic insertion while FLP's potent extrachromosomal array destroying activity ensures that only monomers are inserted by de-concatamerizing insert template DNA which rapidly recombines into large arrays after injection into the germline (Mello et al., 1991). FLP also excises the phiC31 and FLP recombinase cassette sequences after the phiC31 mediated insertion. *jsSi2682* , a single-component landing site with a recombinase cassette linked to the *attP* insertion site, was created using CRISPR and RMHE (see Methods for details). The key features of the landing site are the presence of an *attP* site 5' and a *FRT3* site 3' of the recombinase cassette (Figure 1). Two integration vectors were also created to permit integration into this novel landing site. These vectors contain a *lox2722* flanked drug resistance cassette, an *E. coli* plasmid backbone, an *FRT3* , and an *attB* site along with a Golden Gate cloning compatible multiple cloning site ( Fig. 1A, E).

To create novel transgenes using RMI, a DNA of choice is inserted into either a hyg ^R^ or neo ^R^ integration vector ( Fig. 1A, 1E). In the example shown, a promoter, a fluorescent protein (FP) and a 3' UTR are inserted into the hyg ^R^ vector using a Golden Gate reaction. The resulting plasmid is injected into individual worms, and after one generation hygromycin is added to efficiently select for insertions. Homozygous inserts can be isolated as L4 or adult animals which do not express mNG in the germline (and exhibit any expected visible phenotypes from expression of the insert DNA). Typically, these animals can start to be isolated between day 6 and 9 depending on the robustness of the injection. Initial experiments using this new landing site suggested integration occurs at similar efficiency as previously described RMI landing sites (Nonet, 2024).

To quantify the frequency of insertion, we injected a mixture of 6 different *phat-4 p* FP plasmids, expressing either a nuclear or cytosolic version of a green, an orange and a red fluorescent protein at equal concentration for each plasmid. At each of 4 different concentrations, the DNAs were injected into 8 adult animals and plated individually, and transgenic progeny were characterized ( Fig. 1B ). Injections at several different DNA concentrations yielded transgenes. At high DNA concentration many pre-comma stage arrested embryos were observed which typically appeared yellow-orange likely due to co-expression of the different fluorescent spectral variants. Injections performed at the most optimal DNA concentration (3 ng/ul per construct) yielded resistant progeny in seven of eight injected animals and 3 yielded insertions of all six types. On a typical plate, one to 3 classes of insertions are relatively abundant, and the remaining classes are relatively scarce. This likely is due to both 1) integrations events occurring in early born F1 progeny having a competitive advantage due to birth time and 2) integration events occurring early in germline development contributing more transgene containing germ cells than integration events occurring late in germline development. We believe it is very likely that integration events occur in multiple different F1 progeny, although we have not cloned the progeny of an individual injected animal to directly document this. Furthermore, the isolation of *trans* heterozygotes carrying two distinct FP insertions among the F2 progeny indicates that multiple insertions occur in at least some F1 animals, some of which occur in the spermatogenic germline and others in the oogenic germline. These data are consistent with prior experiments examining the frequency of insertions during RMI (Nonet, 2024).

To confirm that RMI insertions are single copy and of the expected structure, a set of six different insertions (one insertion containing each cytosolic and each nuclear FP) derived from an individual injected P0 animal were characterized in further detail. Genomic DNA was isolated, and the entire insert was amplified using oligonucleotides hybridizing outside of the insertion. The appropriately sized insertion was observed in all six transgenes ( Fig. 1C ; see Extended Data Table for sequence of insertions). PCR products derived from two of the insertions were further subject to nanopore sequencing which confirmed the insertions were of expected sequence.

To document the robustness of the approach, we characterized pharyngeal gland cell promoters. Numerous genes have been previously demonstrated to express relatively specifically in the pharyngeal gland cells (Ghai et al., 2012). However, these studies were all performed using multi-copy extrachromosomal arrays making comparison of the strength of these promoters very difficult. mStayGold fusions to the *phat-2* promoter and six other promoters and Scarlet-I3 fusions to the *phat-2* promoter and six additional promoters were introduced into an RMI vector. Seven pairs of one green and one red fusion construct were each co-injected into 4 animals and placed on two plates. After adding hygromycin, transgenic insertions of both colors were identified on 13 of the 14 plates. This confirmed that all the promoters except *lys-8p* are gland cell specific under the growth conditions we examined, and that most expressed in all gland cells, with *phat-2* and *phat-5* being specific to g1v cells and *gly-15* being specific to g2 gland cells. Multiple other promoters were differentially expressed in the glands cell with some being predominantly expressed in g1d and others in g1v. The promoters also differ in their expression in the g2 gland cells with *srlf-4p* expressing robustly and *phat-1p* expressing very weakly. These fusions provide a set of reliable reporters for pharyngeal gland cells.

The promoters studied herein all express very specifically in pharyngeal gland cells when inserted using a hyg ^R^ selection cassette. However, we have previously observed promoters which are hijacked by the *rps-0* promoter driving hyg ^R^ yielding faint expression of an inserted FP in most tissues. In such cases, the hyg ^R^ cassette can be easily excised by crossing through a germline *Cre* transgenic line (Nonet, 2023). This excision event is typically highly efficient. We recommend using NM6024 *myo-2p Scarlet* marked germline *Cre* strain which is integrated at the same position as the landing site which simplifies identifying homozygous insertion animals when the transgene does not express a visible marker.

RMI provides a new approach to create transgenes. It is particularly useful as a method for creating a set of insertions that differ in one feature that can be visually detected in the transgenic animals. For example, a novel organelle marker could be expressed under the control of a half dozen different promoters, and all six insertions isolated from the injection of a single animal. In practice it makes little sense to inject only one animal as most of the time required to perform an injection experiment is creating and centrifuging the injection mixture, loading a needle with DNA, mounting the needle and establishing flow of the needle. Thus, if a needle does not clog, we typically inject 4 animals distributed on two plates, which we find to be a good compromise between efficiency and time commitment. This eliminates the frustration of the occasional injected animal which dies or crawls off a plate before laying progeny. Furthermore, it allows for the isolation of two independent insertions for each construct.

We have made several attempts to create other single component landing sites. The *jsSi2682* landing site was constructed in three steps. We tried constructing landing sites using RMCE with dual *mex-5* promoters, but these plasmids were not stable in *E. coli* and the sequences between the *mex-5* promoters was deleted. We also tried creating *klp-19 mex-5* dual germline promoter constructs, but in these *klp-19 p::phiC31* fails to express in the germline, despite the fact that *klp-19 p* ::mNG and *mex-5 p* ::mNG constructs integrated independently express well in the germline. We suspect that bidirectional transcription from the *mex-5* promoter may lead to creation of dsRNA and result in silencing of phiC31 expression. We are currently testing *mex-5 p* phiC31 V2A FLP sl2 mNG transgenes. Alternatively, we plan test constructs with divergent germline promoters. If these attempts all fail, we will resort to using a 3-step approach parallel to that used to build *jsSi2682* to build several additional landing sites on other chromosomes.

## Methods

Nomenclature

*C. elegans* RMCE insertions into a landing site locus (e.g., *jsSi2682* ) should technically be called *jsSi#* [* j *sSi2682* ] according to *C. elegans* nomenclature rules (Tuli et al., 2018) but were referred to in the paper as *jsSi#.* A list of strains and a list of transgene insertions is provided in the Extended Data Table. These lists use the a <{region reverse oriented} nomenclature to indicate that a section of the insertion is in the reverse orientation.

*C. elegans* strain maintenance

*C. elegans* was grown on NGM plates seeded with *E. coli* OP50 on 6 cm plates with 10 ml agar. Stocks strains were maintained at RT (~ 22.5°C). (r)RMCE experiments were performed at 25°C.

Molecular Biology

All PCR amplifications used in plasmid and vector constructions were performed using Q5 polymerase (New England Biolabs, Ipswich, MA). Most PCR reactions were performed using the following conditions: 98°C for 0:30, followed by 30 cycles of 98°C for 0:10, 62°C for 0:30, 72°C for 1:00/kb). PCR products were digested with *DpnI* restriction endonuclease to remove template if amplified from a plasmid, then purified using a standard Monarch (New England Biolabs) column purification procedure. The 1 Kb ladder Plus Ladder (Invitrogen) was used as a marker in figures presenting PCR analysis. Restriction enzymes (except for *LguI* ), T4 DNA ligase, and polynucleotide kinase were purchased from New England Biolabs. Golden Gate (GG) reactions (Engler et al., 2008) were performed as described in Nonet (2020) except that in some cases *LguI* (Thermo Scientific™, Waltam, MA) was used in place of SapI. The *E. coli* strain DH5α was used for all transformations. Sanger sequencing was performed by GENEWIZ (South Plainfield, NJ) and nanopore sequencing by Plasmidsaurus (South San Francisco, CA). Oligonucleotides were obtained from MilliporeSigma (Burlington, MA). A detailed description of all constructs, the sequence of all plasmids, and oligonucleotides is provided in Table S1.

Creation of the landing site

To create a single copy transgenic line expressing both phiC31 and FLP, the *bqSi711* transgene (Macías-León and Askjaer, 2018) which expresses both FLP and mNG was modified by the insertion of a 3' *attB* site, and recombined using RMHE with an independently derived *mex-5 p* phiC31 transgene with a 5' *attP* site to create *jsSi1784* (Nonet, 2024). To create an RMI landing site, an *attP* site was integrated on the 5' of *jsSi1784* to create *jsSi2682 .* Specifically, *jsSi2682* was created from NM5641 *jsSi1784* worms by microinjection using a nucleic acid based co-CRISPR strategy with a denatured DNA repair template (Ghanta and Mello, 2020). The *attP* template was amplified from NMp4085 using NMo7852/7853 purified, denatured, and injected at 2ng/ul with 15 ng/ul NM3153 ( *U6p* sgRNA _dpy-10_ ), 43 ng/ul NM5149 ( *U6p* sgRNA _Chr IV_ ), 40 ng/ul NM3143 ( *eft-3p* Cas9), and 500nM oligonucleotide NMo5238 *dpy-10 ( cn64 )* template. Rol animals were allowed to lay eggs, then lysed and analyzed by PCR for insertions using NMo3883/3890. The resulting insertion, *jsSi2682* was outcrossed two times to wild-type animals.

Recombination mediated insertions

DNA was microinjected into worms as previously described (Nonet, 2023). Most animals were only injected in a single gonad. For drug selection, the following were added directly to worm plates 3 days after injection: hyg ^R^ selection - 100 ul of 20 mg/ml hygromycin B (GoldBio, St. Louis, MO), neo ^R^ selection - 500 ul of 25 mg/ml G418 disulfate (Sigma, St. Louis, MO). Six to eight days after injection L4 animals homozygous for insertions could usually be identified on injection plates. For the quantification of insertion frequencies ( Fig. 1B ), hyg ^R^ progeny were scored directly on the dissection microscope as the six FPs can be distinguished on our microscope.

Molecular analysis of insertions

The structure of RMI insertions and the RMI landing site were analyzed using long range PCR of genomic DNA as described in Nonet (2020) using oligonucleotides NMo3889/3890. The sequence of the landing site and two insertions were confirmed using nanopore sequencing. See Table S1 for the sequence of insertions.

Microscopy

Screening of worms for fluorescence during analysis was performed on a Zeiss (White Plains, NY) Stemi SV11 dissecting microscope outfitted with a Lumencor (Beaverton, OR) Sola light source and a Kramer Scientific (Amesbury, MA) M2bio epi-fluorescence module with a long pass GFP filter and an RFP filter and a rotating turret housing both a Zeiss 1.6X objective and a Kramer 10X (n.a. 0.45) objective for high power observation. For imaging or quantification of fluorescence, worms were mounted on 2% agarose pads in a 2 µl drop of 1 mM levamisole in phosphate buffered saline. Ten to twenty L4 animals were typically placed on a single slide. Animals were imaged on an Olympus (Center Valley, PA) BX-60 microscope run using Micro-Manager 2.0ß software (Edelstein et al., 2014) using either a 20X air (n.a. 0.5) or 40X air (n.a. 0.75) plan-NEOFLUAR objective. The scope is equipped with a ToupTek MAX04BM sCMOS camera, a Lumencor AURA LED light source, an Applied Scientific Instruments (Eugene, OR) MS-4000 XY piezo Z motorized stage, Ludl (Hawthorne, NY) high speed electronic filter wheels and shutters and a Chroma 59022 eGFP/MCherry dual band filter set. Images were quantified using the FIJI version of ImageJ software (Schindelin et al., 2012) as described in Nonet (2020). To quantify gland cell signals, a 100 pixel diameter circle centered over the posterior bulb of the pharynx was used as the ROI. Background was subtracted using an equal sized circle ROI placed elsewhere in the field. Data plots were created using PlotsOfData (Postma and Goedhart, 2019).

## Reagents

**Table:** 

Strain Name	genotype	Source
NM5641	*jsSi1784 [ mex-5 p::FLP::sl2::mNG Cbunc-119(+) mex-5 Kp phiC31] IV *	Nonet, 2024
NM6907	*jsSi2682 [attP mex-5 p::FLP::sl2::mNG Cbunc-119(+) mex-5 Kp phiC31] IV *	this study
NM6927	*jsSi2682 [attP mex-5 p::FLP::sl2::mNG Cbunc-119(+) mex-5 Kp phiC31] IV* 2X backcrossed	this study
NM6920	*jsSi2693 [HygR phat-4 p nls-cyOFP] IV *	this study
NM6921	*jsSi2694 [HygR phat-4 p nls-nNG] IV *	this study
NM6923	*jsSi2695 [HygR phat-4 p nls-Scarlet] IV *	this study
NM6924	*jsSi2696 [HygR phat-4 p cyOFP] IV *	this study
NM6922	*jsSi2697 [HygR phat-4 p mStaygold] IV *	this study
NM6925	*jsSi2698 [HygR phat-4 p mScarlet] IV *	this study
NM6930	*jsSi2703 [HygR phat-2 p Scarlet-I3] IV *	this study
NM6935	*jsSi2704 [HygR ZK596.1p Staygold] IV *	this study
NM6931	*jsSi2705 [HygR scl-6p Scarlet-I3] IV *	this study
NM6932	*jsSi2706 [HygR srlf-4p Staygold] IV *	this study
NM6941	*jsSi2712 [HygR phat-4 p Scarlet-I3] IV *	this study
NM6942	*jsSi2713 [HygR R05G6.9p Staygold] IV *	this study
NM6943	*jsSi2714 [HygR B0280.7p Scarlet-I3] IV *	this study
NM6944	*jsSi2715 [HygR dod-6p Staygold] IV *	this study
NM6945	*jsSi2716 [HygR tag-293p Scarlet-I3] IV *	this study
NM6946	*jsSi2717 [HygR phat-1p Staygold] IV *	this study
NM6947	*jsSi2719 [HygR lys-8p Scarlet-I3] IV*	this study
NM6949	*jsSi2720 [HygR phat-5 p Staygold* ] *IV*	this study
NM6948	*jsSi2721 [HygR phat-2 p Staygold] IV *	this study
NM6960	*jsSi2722 [HygR gly-15 p Scarlet-I3] IV *	this study

Worm strains and plasmids are available by request from MLN. The chr IV landing site is available from the *Caenorhabditis* Genetics Center. The two RMI vectors have been submitted to Addgene.

## Data Availability

Description: DNA constructs, oligonucleotides, Transgenes, alleles, Strain and Plasmid construction methods. Resource Type: Text. DOI: https://doi.org/10.22002/cvjz8-afh27

## References

[R1] Altun ZF, Hall DH. 2020. Handbook of *C. elegans* Anatomy. Worm Atlas Available: http://www.wormatlas.org via the Internet.

[R2] Edelstein AD, Tsuchida MA, Amodaj N, Pinkard H, Vale RD, Stuurman N (2014). Advanced methods of microscope control using μManager software.. J Biol Methods.

[R3] Ghai V, Smit RB, Gaudet J (2012). Transcriptional regulation of HLH-6-independent and subtype-specific genes expressed in the Caenorhabditis elegans pharyngeal glands.. Mech Dev.

[R4] Ghanta KS, Mello CC (2020). Melting dsDNA Donor Molecules Greatly Improves Precision Genome Editing in
*Caenorhabditis elegans*
.. Genetics.

[R5] Knoebel E, Brinck A, Nonet ML (2025). Parameters that influence bipartite reporter system expression in Caenorhabditis elegans.. Genetics.

[R6] Macías-León J, Askjaer P (2018). Efficient FLP-mediated germ-line recombination in
*C. elegans*
.. MicroPubl Biol.

[R7] Mello CC, Kramer JM, Stinchcomb D, Ambros V (1991). Efficient gene transfer in C.elegans: extrachromosomal maintenance and integration of transforming sequences.. EMBO J.

[R8] Nonet ML (2020). Efficient Transgenesis in
*Caenorhabditis elegans*
Using Flp Recombinase-Mediated Cassette Exchange.. Genetics.

[R9] Nonet ML (2023). Rapid generation of Caenorhabditis elegans single-copy transgenes combining recombination-mediated cassette exchange and drug selection.. Genetics.

[R10] Nonet Michael L. (2024). Creation and manipulation of bipartite expression transgenes in
*C. elegans*
using phiC31 recombinase.

[R11] Postma M, Goedhart J (2019). PlotsOfData-A web app for visualizing data together with their summaries.. PLoS Biol.

[R12] Schindelin J, Arganda-Carreras I, Frise E, Kaynig V, Longair M, Pietzsch T, Preibisch S, Rueden C, Saalfeld S, Schmid B, Tinevez JY, White DJ, Hartenstein V, Eliceiri K, Tomancak P, Cardona A (2012). Fiji: an open-source platform for biological-image analysis.. Nat Methods.

[R13] Tuli MA, Daul A, Schedl T (2018). Caenorhabditis nomenclature.. WormBook.

